# Association between relative fat mass and rheumatoid arthritis: A cross-sectional study

**DOI:** 10.1097/MD.0000000000047822

**Published:** 2026-03-06

**Authors:** Yuanting Sun, Ruiji Wu, Yanze Lin

**Affiliations:** a Fuyang TCM Hospital of Orthopedics Affiliated to Zhejiang Chinese Medical University (Hangzhou Fuyang Hospital of Orthopedics of Traditional Chinese Medicine), Hangzhou, Zhejiang, China; b Department of Orthopaedics, Taizhou Hospital of Zhejiang Province Affiliated to Wenzhou Medical University, Linhai, Zhejiang, China; c Department of Orthopaedics, The Third People’s Hospital of Yuhang District, Hangzhou, Zhejiang, China.

**Keywords:** cross-sectional study, NHANES, relative fat mass, rheumatoid arthritis, ROC

## Abstract

Obesity has been recognized as a significant contributor to the development of rheumatoid arthritis (RA). Relative fat mass (RFM), a newly proposed anthropometric index, offers an alternative method for estimating body fat percentage. This cross-sectional study aims to elucidate the potential association between RFM and RA risk. Data were obtained from the National Health and Nutrition Examination Survey 1999 to 2018. Weighted multivariable logistic regression was used to assess the independent association between RFM and RA, and subgroup interaction analyses were performed to evaluate potential effect modification. Restricted cubic spline (RCS) models were applied to explore potential nonlinear relationships. Receiver operating characteristic (ROC) curve analysis was conducted to compare the discriminatory ability of RFM and body mass index (BMI). This cross-sectional study demonstrates a positive association between RFM and RA. Although RFM offers some improvement over BMI as an obesity-related indicator, its clinical utility for identifying RA risk remains limited. A total of 43,499 participants were included in the analysis, among whom 2557 had RA. Weighted multivariable logistic regression revealed a significant positive association between RFM and RA (odds ratio = 1.048; 95% confidence interval: 1.036–1.060). This association remained consistent when RFM was analyzed in quartiles, with individuals in the highest quartile showing a significantly higher risk of RA compared to the lowest quartile (odds ratio = 2.487; 95% confidence interval: 1.875–3.299). Subgroup interaction analyses revealed that this association was modified by age, educational level and smoking status. RCS analysis demonstrated a linear relationship between RFM and RA. RFM showed slightly better discrimination than BMI in ROC analysis, but the overall discriminatory performance remained modest (area under the curve = 0.614 vs 0.581).

## 1. Introduction

Rheumatoid arthritis (RA) is a prevalent chronic systemic autoimmune disease, primarily characterized by symmetrical polyarthritis, typically affecting the small joints of the hands and feet, but also capable of involving large joints and multiple organ systems.^[1,2]^ Its key pathological features include chronic synovitis, pannus formation, and progressive destruction of articular cartilage and subchondral bone, ultimately resulting in joint deformities, functional impairment, and irreversible disability.^[3,4]^ The etiology of RA is multifactorial, involving the interplay of genetic susceptibility, environmental exposures (such as smoking, obesity, and gut microbiota dysbiosis), and dysregulated immune responses.^[5–7]^ Epidemiologically, an estimated 0.5% to 1% of the global population is affected by RA, with a significantly higher prevalence in women, and it can affect individuals of all ages.^[8,9]^ Despite advancements in biologic therapies and targeted synthetic agents that have improved disease management and facilitated treat-to-target strategies, a substantial proportion of patients continue to exhibit inadequate treatment responses or develop therapeutic resistance.^[10]^ Consequently, RA remains a major public health challenge associated with significant societal and economic burdens.^[11]^ These considerations underscore the importance of improving early screening, diagnostic evaluation, and individualized treatment approaches.

Obesity has increasingly been recognized as a modifiable factor contributing to systemic inflammation and immune dysregulation, and growing evidence suggests a positive relationship between obesity and RA risk.^[12–16]^ Prior epidemiologic studies have demonstrated that higher body mass index (BMI), waist circumference (WC), and body fat percentage are associated with increased RA incidence or prevalence. Meta-analytic findings indicate a dose–response relationship between BMI and RA risk,^[17]^ while cohort studies have shown that central adiposity and increased body fat percentage may further contribute to RA development.^[18]^ Collectively, these observations highlight the importance of accurately assessing adiposity when evaluating RA susceptibility.

However, BMI, though widely used in clinical and epidemiologic settings, has well-recognized limitations. It does not differentiate between fat and lean mass, nor does it reflect sex-specific or regional fat distribution, potentially leading to misclassification in obesity-related epidemiologic research. To overcome these limitations, several alternative anthropometric indicators have been proposed, including the weight-adjusted waist index and body roundness index, both of which have demonstrated improved performance in predicting metabolic disorders.^[19,20]^ Among these emerging metrics, relative fat mass (RFM) has gained particular attention. RFM is a novel index for estimating total body fat percentage based on height-to-waist-circumference ratios with sex-specific adjustments.^[21]^ However, no studies to date have investigated the association between RFM and RA risk, and its applicability in RA screening or risk prediction remains unexplored.

Therefore, this study aims to investigate the association between RFM and RA using data from the National Health and Nutrition Examination Survey (NHANES) 1999 to 2018. Additionally, it evaluates whether RFM provides additional value beyond BMI in assessing RA risk, with the goal of offering new insights and evidence-based support for improving early identification and precision prevention of RA.

## 2. Methods

### 2.1. Study population

The data for this study were derived from the NHANES, a program administered by the U.S. Centers for Disease Control and Prevention. NHANES utilizes a stratified, multistage probability sampling methodology to obtain nationally representative estimates of the health and nutritional status of the noninstitutionalized U.S. population. Data collection involves standardized interviews, physical examinations, and laboratory testing, all of which are conducted at designated Mobile Examination Centers (MECs). All survey protocols received ethical approval from the Ethics Review Board of the National Center for Health Statistics, with written consent secured from each participant. NHANES data are publicly available and have been extensively used in epidemiological studies, health risk evaluation, and public health policy development. The datasets analyzed in this study can be accessed via the official NHANES website (https://www.cdc.gov/nchs/nhanes.htm).

**Figure 1.**
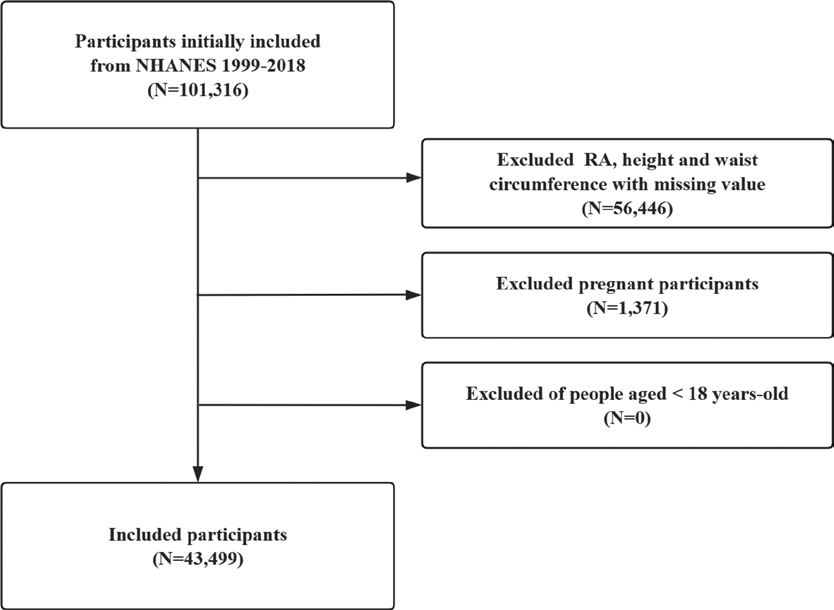
Participant selection flowchart for the study cohort.

In this study, we initially included 101,316 participants across ten consecutive NHANES cycles. The following exclusion criteria were applied: missing data on RA status, height, or WC; pregnant individuals; age younger than 18 years. Finally, a total of 43,499 participants remained eligible for the final analysis (Fig. 1).

### 2.2. RA diagnosis

The diagnosis of RA was established based on self-reported questionnaire data from NHANES. Participants were initially asked, “Has a doctor or other health professional ever told you that you had arthritis?” Those who responded affirmatively were subsequently asked to specify the type of arthritis diagnosed. Only individuals who explicitly identified “rheumatoid arthritis” were classified as RA cases and included in the analysis. Previous research has demonstrated that self-reported RA based on questionnaire responses is highly consistent with clinically confirmed diagnoses.^[22]^

### 2.3. RFM calculation

RFM is an anthropometric index used to assess obesity status, calculated based on measurements of height and WC. These 2 parameters were measured by trained health technicians following standardized protocols at the MECs. RFM was calculated using the following sex-specific equations: RFM = 64 − (20 × height/WC) for males, and RFM = 76 − (20 × height/WC) for females.^[23]^

### 2.4. Covariates

Drawing on prior research, the following covariates were incorporated into the analysis: gender, age, race, education, poverty income ratio (PIR), marital status, smoking status, alcohol consumption. Age was stratified into 3 groups: 20 to 39, 40 to 59, and 60 years or older. Race was classified into 5 categories: Mexican American, other Hispanic, Non-Hispanic Black, Non-Hispanic White, and other race. Educational level was grouped as less than high school, high school, and more than high school. PIR was divided into 3 levels: <1.3, 1.3 to 3.5, and > 3.5. Marital status was simplified as living alone or living with others. Smoking was divided into never, former and current smokers. Alcohol consumption was classified into five gradations: never, former, light, moderate, and heavy drinkers, based on frequency and quantity of intake.^[24]^

### 2.5. Handling of missing variables

To preserve the largest possible sample size, multiple imputation was employed to address missing variables, despite the relatively small proportion of missing data. The distribution of variables with missing data in our study is illustrated in Fig. S1, Supplemental Digital Content, https://links.lww.com/MD/R448.

### 2.6. Statistical analysis

Given the complex, multistage probability sampling design of NHANES, appropriate sample weights were applied in all statistical analyses to produce nationally representative estimates. Specifically, for the 1999 to 2000 and 2001 to 2002 cycles, the sample weight was calculated as 2/10 × WTMEC4YR, whereas for the 2003 to 2018 cycles, it was calculated as 1/10 × WTMEC4YR. In the analysis of baseline characteristics, normally distributed continuous variables were presented as mean ± standard deviation, whereas non-normally distributed continuous variables were reported as median and interquartile range. Categorical variables were expressed as percentages. To compare baseline characteristics between the RA and non-RA groups, appropriate statistical tests were applied according to the type and distribution of the variables: 1-way analysis of variance for normally distributed variables, the Kruskal–Wallis test for non-normally distributed variables, and Pearson chi-square test for categorical variables.

To examine the relationship between RFM and the risk of RA, multivariable logistic regression analyses were conducted. We assessed multicollinearity among the independent variables by calculating the variance inflation factor to check the assumptions of logistic regression. No violations of the logistic regression assumptions were detected. RFM was stratified into quartiles (Q1–Q4), with the lowest quartile (Q1) designated as the reference category. Three models were developed to incrementally adjust for potential confounders. Model 1 was unadjusted; Model 2 adjusted for gender, age, and race; and Model 3 included further adjustments for education level, PIR, marital status, smoking status, and alcohol consumption. To assess potential effect modification, subgroup interaction analyses were conducted across different population strata. In addition, the restricted cubic spline (RCS) analysis was applied to assess the potential nonlinear relationship. Finally, receiver operating characteristic (ROC) curves analysis was used to assess and compare the predictive accuracy of RFM and BMI for RA, with performance quantified by the area under the curve (AUC).

All statistical analyses were conducted using R software (version 4.4.0). A 2-sided *P*-value was used to determine statistical significance, with *P* < .05 considered statistically significant.

### 2.7. Sensitivity analysis

We conducted a sensitivity analysis by excluding participants with missing data on any study variables. Logistic regression models were then refitted to reevaluate the robustness of the study findings (Table S1, Supplemental Digital Content, https://links.lww.com/MD/R448).

## 3. Results

### 3.1. Baseline characteristics

Table 1 summarizes the baseline characteristics of the study population. Among the 43,499 participants included, the overall prevalence of RA was 5.9%. Compared with individuals without RA, those with RA were older and more likely to be female, and a higher proportion were non-Hispanic Black. Participants with RA also exhibited lower educational attainment, lower socioeconomic status, and were more likely to live alone. With respect to lifestyle factors, the RA group had higher prevalences of ever and current smoking and a greater proportion of former drinkers, whereas moderate and heavy alcohol consumption was less common. Additionally, participants with RA had significantly higher BMI and RFM. All differences were statistically significant (*P* < .05).

**Table 1:** Characteristics of study participants in NHANES 1999 to 2018.

Characteristics	Overall (N = 43,499)	None-RA (N = 40,942)	RA (N = 2557)	*P*-value
Age (years)	46.02 ± 16.55	45.50 ± 16.46	57.24 ± 14.31	<.001
Gender (%)				<.001
Male	49.35	49.70	41.86	
Female	50.65	50.30	58.14	
Race (%)				<.001
Mexican American	8.34	8.43	6.36	
Other Hispanic	5.68	5.72	4.91	
Non-Hispanic White	68.04	68.11	66.55	
Non-Hispanic Black	10.92	10.66	16.47	
Other race	7.02	7.08	5.71	
Education level (%)				<.001
Lower than high school	16.38	15.95	25.76	
High school	23.81	23.61	28.17	
More than high school	59.81	60.44	46.07	
PIR (%)				<.001
Below 1.3	21.10	20.65	30.90	
1.3–3.5	36.04	35.98	37.24	
Over 3.5	42.86	43.36	31.86	
Marital status (%)				.005
Live alone	36.10	35.96	39.11	
Live with others	63.90	64.04	60.89	
Smoking status (%)				<.001
Never	54.20	54.81	41.07	
Ever	24.05	23.69	31.78	
Current	21.75	21.50	27.15	
Alcohol consumption (%)				<.001
Never drinker	11.07	10.98	13.06	
Former drinker	15.05	14.53	26.11	
Light	35.60	35.74	32.55	
Moderate	17.01	17.21	12.92	
Heavy	21.27	21.54	15.36	
BMI (kg/m^2^)	28.55 ± 6.59	28.47 ± 6.55	30.21 ± 7.29	<.001
RFM (mean ± SD)	34.70 ± 8.48	34.55 ± 8.46	37.83 ± 8.20	<.001

Continuous variables were presented as mean ± SD. Categorical variables were presented as n (%).

BMI = body mass index, NHANES = National Health and Nutrition Examination Survey, PIR = poverty-to-income ratio, RA = rheumatoid arthritis, RFM = relative fat mass.

### 3.2. Relationship between RFM and RA

The weighted multivariable logistic regression analysis demonstrated a significant positive correlation between RFM and the risk of RA (Table 2). This relationship remained statistically significant across all models, including the unadjusted Model 1 (odds ratio, OR = 1.047; 95% confidence interval, CI: 1.040–1.054), the partially adjusted Model 2 (OR = 1.051; 95% CI: 1.038–1.063), and the fully adjusted Model 3 (OR = 1.048; 95% CI: 1.036–1.060). When RFM was analyzed as quartiles, participants in higher quartiles consistently exhibited greater odds of RA compared with those in the lowest quartile across all models. In the fully adjusted model, the odds of RA increased progressively from the second quartile (OR = 1.544, 95% CI: 1.251–1.906) to the fourth quartile (OR = 2.487, 95% CI: 1.875–3.299). A significant dose–response relationship was observed, as indicated by a significant trend across RFM quartiles in all models (*P* for trend < 0.001).

**Table 2:** Association between RFM and RA.

	Model 1 (95% CI) *P*-value	Model 2 (95% CI) *P*-value	Model 3 OR (95% CI) *P*-value
RFM	1.047 (1.040–1.054) <.001	1.051 (1.038–1.063) <.001	1.048 (1.036–1.060) <.001
Q1 (7.756–28.722)	Reference	Reference	Reference
Q2 (28.722–34.324)	1.807 (1.479–2.208) <.001	1.502 (1.221–1.847) <.001	1.544 (1.251–1.906) <.001
Q3 (34.324–42.149)	1.780 (1.463–2.167) <.001	1.679 (1.308–2.155) <.001	1.698 (1.320–2.183) <.001
Q4 (42.149–58.412)	3.063 (2.542–3.690) <.001	2.598 (1.959–3.445) <.001	2.487 (1.875–3.299) <.001
*P* for trend	<.001	<.001	<.001

Model 1: No covariates were adjusted.

Model 2: Adjusted for age, gender, and race.

Model 3: Adjusted for age, gender, race, education level, marital status, ratio of family income to poverty, smoking status, and alcohol consumption.

CI = confidence interval, OR = odds ratio, RA = rheumatoid arthritis, RFM = relative fat mass.

### 3.3. Subgroup interaction analysis

**Figure 2.**
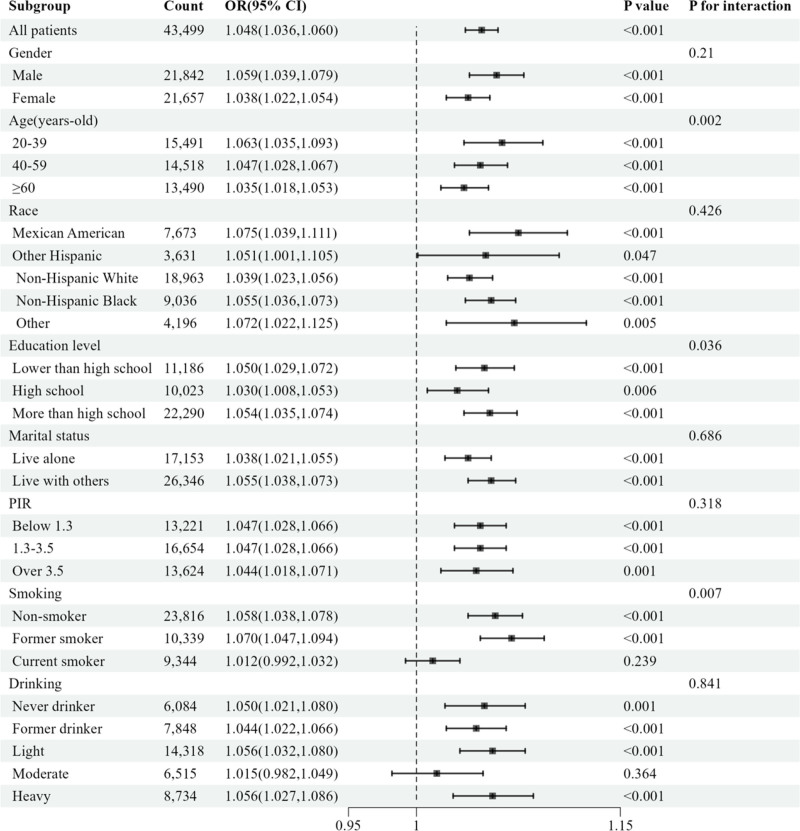
Subgroup analysis and interaction test of the association between the RFM and RA risk. CI = confidence interval, OR = odds ratio, PIR = poverty income ratio, RA = rheumatoid arthritis, RFM = relative fat mass.

To assess whether the association between RFM and RA differed among specific subgroups, a weighted subgroup interaction analysis was conducted. The results indicated that the relationship was not statistically significant in certain subgroups, including current smokers, and moderate drinkers. Notably, the interaction analysis suggested that the association between RFM and RA was significantly modified by age, education level and smoking status (*P* for interaction < .05). Detailed subgroup-specific results are presented in Fig. 2.

### 3.4. RCS analysis

**Figure 3.**
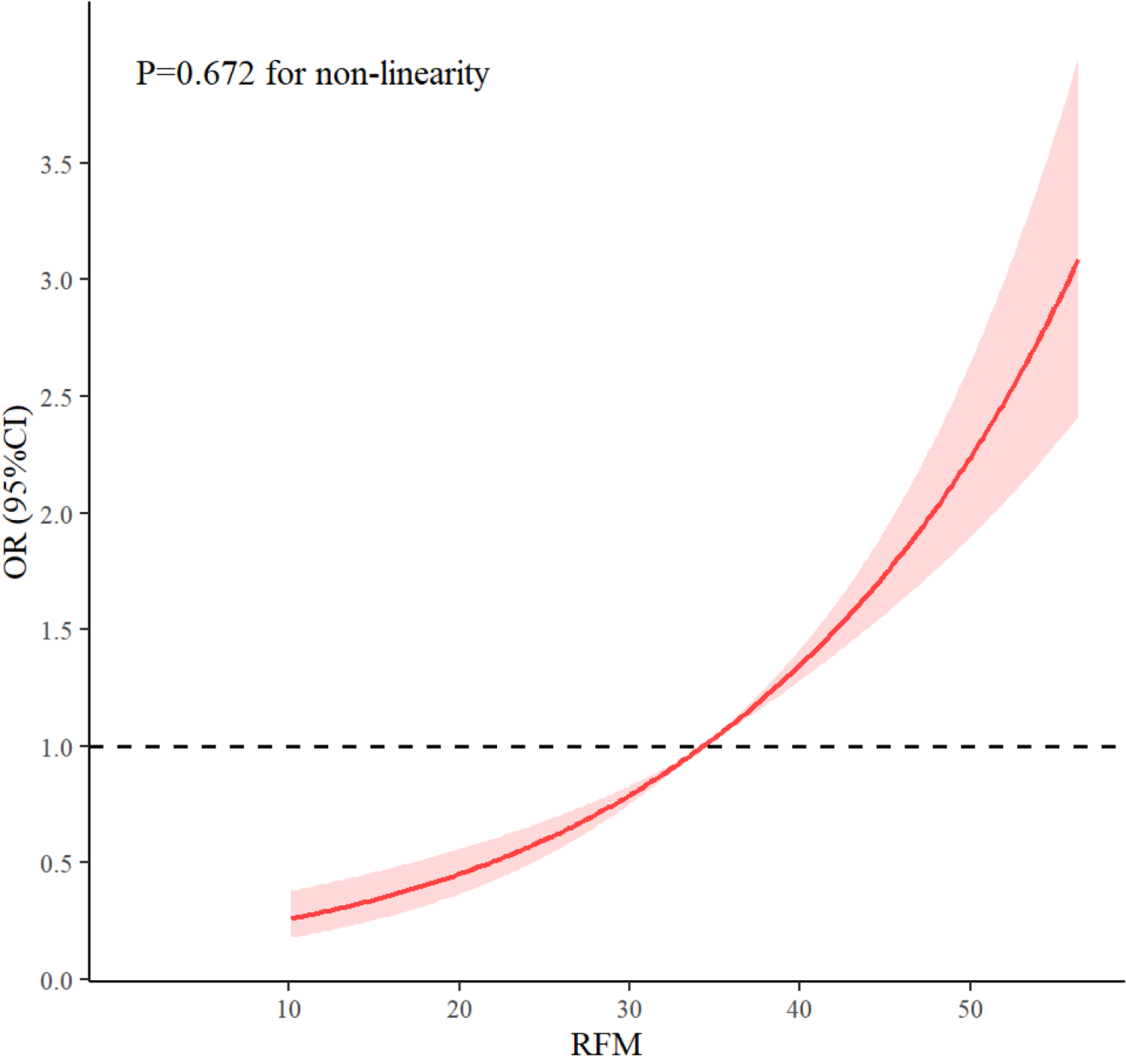
RCS analysis of RFM and the RA. The solid red line indicates the fitted curve between RFM and RA risk, while the light red-shaded area indicates the 95% CI for the curve. The analysis was adjusted for age, gender, race, education level, poverty income ratio, marital status, smoking status, and alcohol consumption. CI = confidence interval, OR = odds ratio, RA = rheumatoid arthritis, RFM = relative fat mass.

A weighted RCS analysis was conducted to evaluate the potential nonlinear relationship between RFM and RA. As illustrated in Fig. 3, RFM showed a positive association with RA risk using a 3-knot model. The nonlinearity test yielded a nonsignificant result (*P* for nonlinearity = 0.672), indicating that the relationship is linear. Meanwhile, the fitted curve remained relatively flat at lower RFM levels, indicating minimal changes in RA risk, whereas a progressively steeper increase in risk was observed at higher RFM levels. To validate the robustness of this finding, additional RCS models incorporating 4 and 5 knots were examined. The results remained consistent, demonstrating a stable linear association between RFM and RA with a persistent positive trend (Figs. S2 and S3, Supplemental Digital Content, https://links.lww.com/MD/R448).

### 3.5. ROC results

**Figure 4.**
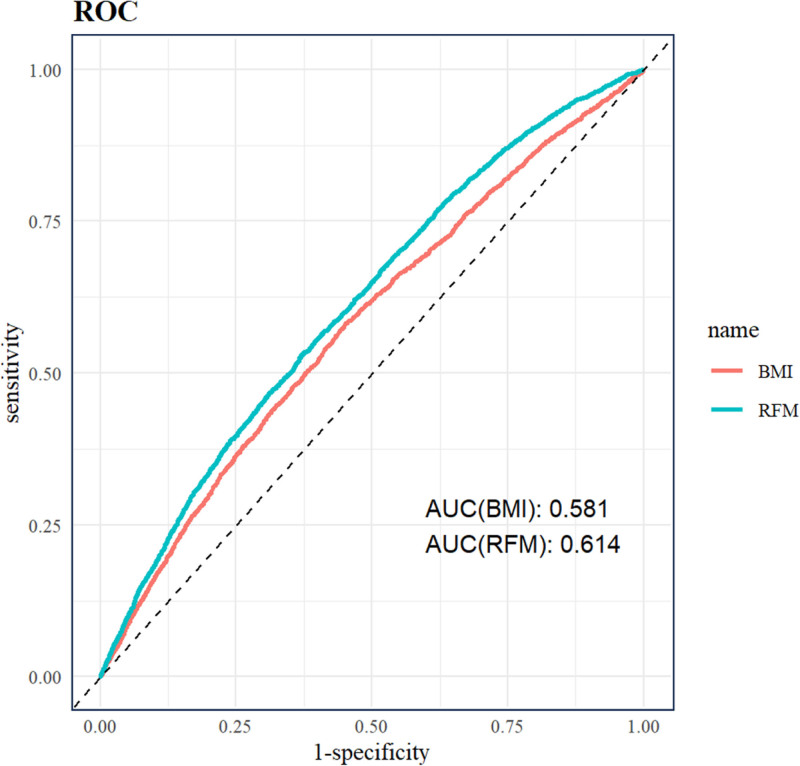
ROC curves of RFM and RA. AUC = area under the curve, BMI = body mass index, RA = rheumatoid arthritis, RFM = relative fat mass, ROC = receiver operating characteristic.

To evaluate the discriminatory capacity of RFM as a predictive indicator for RA, we plotted the ROC curve (Fig. 4) and calculated the AUC. RFM achieved an AUC of 0.614, which was higher than that of BMI (AUC = 0.581), suggesting that RFM has relatively better discrimination for identifying individuals at risk of RA. To further assess the robustness of this finding, we conducted bootstrap resampling of the ROC analysis. Statistical comparisons confirmed that the AUC for RFM was significantly greater than that for BMI (Table S2, Supplemental Digital Content, https://links.lww.com/MD/R448). Nevertheless, the discriminatory performance of both indices remains modest, indicating limited clinical utility (AUC < 0.7).

## 4. Discussion

Using NHANES data from 1999 to 2018, we conducted a large-scale cross-sectional analysis to examine relationship between RFM and RA. The findings showed a significant positive association between higher RFM and an elevated risk of RA. Subgroup interaction analyses further revealed this association was influenced by age, educational level and smoking status. Moreover, RCS analysis demonstrated a linear positive correlation between RFM and RA. ROC curve analysis showed that RFM demonstrated better discriminatory performance than BMI; however, its overall ability to distinguish RA risk remained limited (AUC < 0.7).

RA significantly compromises patients’ quality of life and their ability to perform daily activities.^[25]^ Obesity has been increasingly recognized as an important comorbidity associated with RA. Previous studies using NHANES data and other population-based cohorts have reported significant associations between RA and traditional obesity-related measures, including BMI, WC, and body fat mass assessed by dual-energy X-ray absorptiometry (DXA).^[17,18,26,27]^ These findings emphasize the importance of accurately assessing obesity in preventing and managing RA. However, existing indicators have notable limitations in practical application. For example, BMI fails to distinguish between fat and muscle mass; WC reflects only abdominal fat accumulation; and although DXA provides accurate measurements of body distribution, its high cost and limited accessibility restrict its widespread use. In contrast, RFM, a metric derived from simple anthropometric parameters to estimate body fat percentage, offers distinct advantages such as ease of use and broad applicability.^[21]^ RFM has demonstrated promising utility in the context of various chronic diseases. For instance, a large-scale population-based study among U.S. adults identified a strong association between RFM and colorectal cancer.^[28]^ Another investigation found RFM to be more sensitive than conventional methods in predicting the risk of psoriasis.^[29]^ Additionally, elevated RFM levels have been negatively correlated with cognitive function in older men, suggesting that monitoring and managing RFM may contribute to delaying cognitive decline in the elderly.^[30]^ However, no studies have yet specifically explored the relationship between RFM and RA, highlighting an urgent need for further investigation in this area.

Although the mechanisms linking obesity to RA are not fully defined, accumulating evidence indicates that obesity actively contributes to RA pathogenesis.^[12]^Adipose tissue, particularly visceral fat, functions as an immunometabolically active organ that secretes pro-inflammatory adipokines, including leptin, resistin, and visfatin, which enhance cytokine production and inflammatory signaling via NF-κB and JAK/STAT pathways.^[31–34]^ Certain adiponectin isoforms may also exert pro-inflammatory effects in RA. Collectively, adipose-derived factors promote a chronic low-grade inflammatory state that favors autoimmune activation.^[35]^ Obesity further disrupts immune homeostasis by driving M1 macrophage polarization, impairing regulatory T-cell function, and enhancing B-cell activation, leading to increased production of pathogenic autoantibodies such as anticitrullinated protein antibodies and subsequent synovial inflammation.^[36–40]^ In parallel, obesity-induced gut barrier dysfunction and dysbiosis facilitate systemic exposure to microbial products, while metabolic abnormalities characteristic of metabolic syndrome amplify oxidative stress and endothelial activation, collectively reinforcing systemic inflammation and RA susceptibility.^[41–45]^ At the epigenetic and structural levels, obesity induces persistent reprogramming of immune responses through altered microRNA expression and DNA methylation, skews mesenchymal stem cell differentiation toward adipogenesis, and impairs bone remodeling.^[46,47]^ In addition, increased adipose-derived estrogen production, particularly in women, may further enhance B-cell survival and autoantibody generation.^[48]^ Ultimately, obesity is intricately linked with various psychosocial stressors, particularly depression, which not only frequently co-occurs with obesity but has also been independently linked to the risk of developing RA.^[49–51]^

According to commonly accepted standards for model discrimination, an AUC value below 0.7 is generally considered to indicate poor discriminatory ability. In the present study, although RFM showed a slightly higher AUC than BMI, the AUC values for both indices remained below the threshold for acceptable discrimination. This indicates that, while RFM may offer modest improvement over BMI in distinguishing individuals with RA, its standalone ability to discriminate RA status is limited.

Our study possesses several strengths. Firstly, we introduced a novel obesity-related indicator to explore its association with RA, which has not been reported in previous research. This indicator accounts for fat distribution and sex-specific differences, overcoming the limitations of traditional measures. Secondly, the use of a large, nationally representative sample strengthens the statistical power and generalizability. Thirdly, the multivariable logistic regression analysis clearly revealed a robust association between this indicator and RA, which was further supported by RCS analysis indicating a linear relationship. Moreover, the ROC curve results demonstrated the superior performance of this indicator in assessing RA. Finally, subgroup analyses suggested that age, educational level and smoking status may significantly modify the relationship between obesity and RA, offering novel perspectives for targeted prevention strategies and future mechanistic studies.

Several limitations of this study warrant consideration. First, the cross-sectional design precludes the establishment of causal relationships and raises the possibility of reverse causation. Specifically, RA is characterized by chronic pain, joint damage, and functional limitations, which may reduce physical activity and mobility, thereby promoting weight gain and increased adiposity. Consequently, the higher RFM observed among individuals with RA may partly reflect disease-related changes rather than a unidirectional effect of adiposity on RA development. Second, RA diagnosis relied solely on self-reported questionnaires, which is known to have limited diagnostic accuracy in NHANES. Self-reported RA may result in misclassification, either overestimating or underestimating true prevalence, and may not reliably capture disease severity. Such limitations introduce potential recall bias and could bias the observed associations, potentially attenuating or exaggerating the relationship between adiposity and RA. Third, the NHANES dataset lacks information on RA seropositivity (e.g., rheumatoid factor or anticitrullinated protein antibodies status), limiting our ability to perform stratified analyses based on disease subtypes. Fourth, our study population was restricted to individuals in the United States, which may limit the generalizability of the results to other populations. Therefore, validation in more diverse cohorts is needed. Finally, the observed associations require further investigation through longitudinal studies and experimental or clinical research to elucidate the underlying biological mechanisms.

## 5. Conclusion

In conclusion, RFM was associated with RA in a cross-sectional NHANES sample and demonstrated slightly better discriminatory ability than BMI, although its overall predictive performance was limited. Further validation in prospective cohort studies, along with mechanistic investigations, is warranted to clarify the temporal relationship and biological pathways underlying this association and to assess its generalizability across diverse populations.

## Acknowledgments

The authors would like to extend their appreciation to all the participants of the NHANES.

## Author contributions

**Conceptualization:** Yanze Lin.

**Data curation:** Yuanting Sun, Ruiji Wu.

**Formal analysis:** Yuanting Sun, Ruiji Wu.

**Investigation:** Ruiji Wu.

**Methodology:** Yuanting Sun, Ruiji Wu, Yanze Lin.

**Project administration:** Yanze Lin.

**Software:** Yuanting Sun, Ruiji Wu, Yanze Lin.

**Validation:** Ruiji Wu, Yanze Lin.

**Writing – original draft:** Yuanting Sun.

**Writing – review & editing:** Ruiji Wu, Yanze Lin.

## Supplementary Material


