# Supplementary material for: Association between relative fat mass and rheumatoid arthritis: A cross-sectional study
Source: Medicine (Baltimore). 2026 Mar 6;105(10):e47822. doi: 10.1097/MD.0000000000047822 (PMC12975257; doi:10.1097/MD.0000000000047822)
Supplement: Supplementary file 1 [file medi-105-e47822-s001.docx]

Fig.S1 The distribution of variables with missing data. PIR: Poverty income ratio; BMI:Body mass index; RA: Rheumatoid arthritis; RFM=Relative fat mass.

**
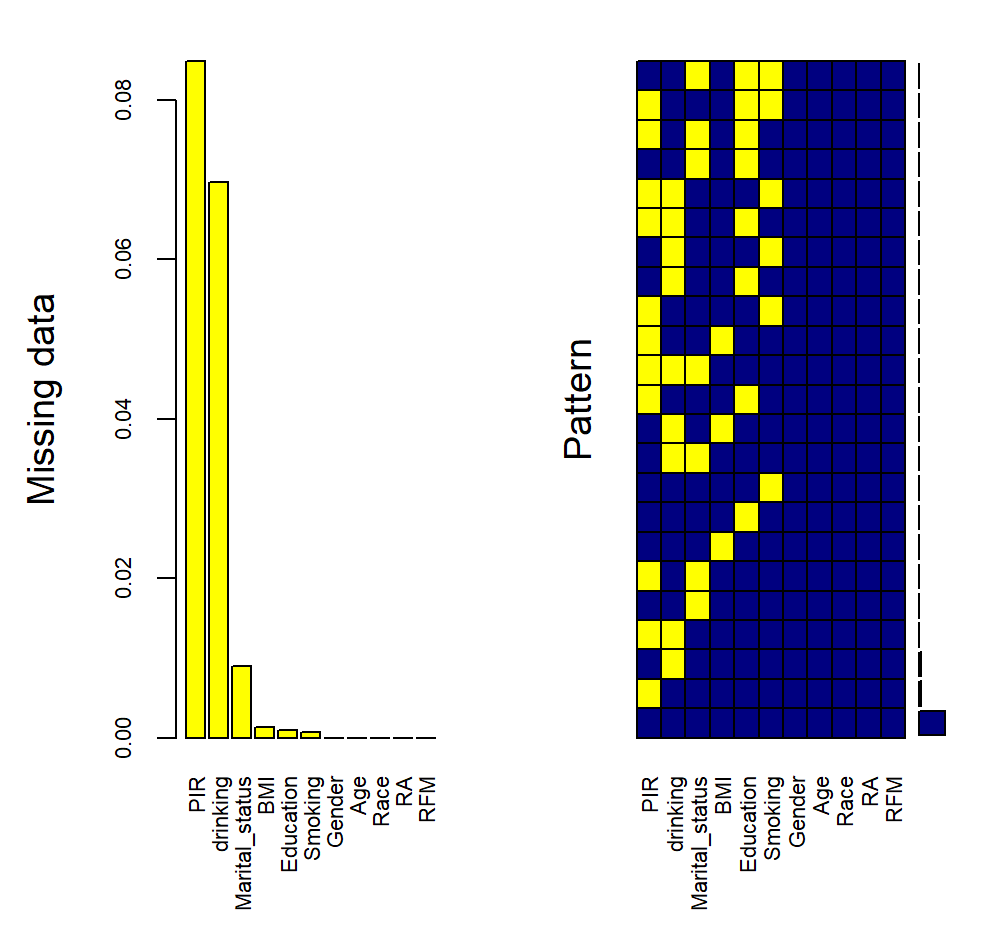
**

Fig.S2 RCS results of the association between RFM and RA(Knot=4). The solid red line indicates the fitted curve between RFM and RA risk, while the light red shaded area indicates the 95 percent confidence interval for the curve. The analysis was adjusted for age, gender, race, education level, poverty income ratio, marital status, smoking status, alcohol consumption. OR=Odds ratio; CI=Confidence interval; RFM=Relative fat mas
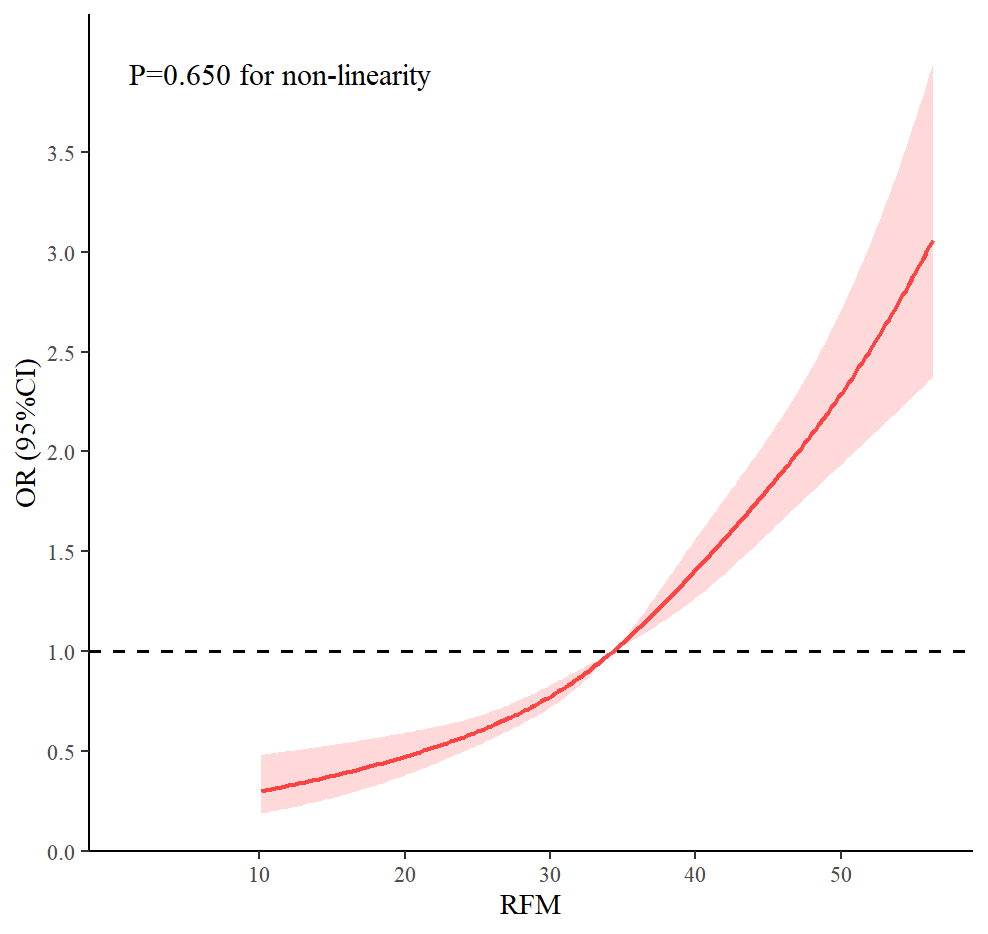


Fig.S3 RCS results of the association between RFM and RA(Knot=5). The solid red line indicates the fitted curve between RFM and RA risk, while the light red shaded area indicates the 95 percent confidence interval for the curve. The analysis was adjusted for age, gender, race, education level, poverty income ratio, marital status, smoking status, alcohol consumption. OR=Odds ratio; CI=Confidence interval; RFM=Relative fat mass.


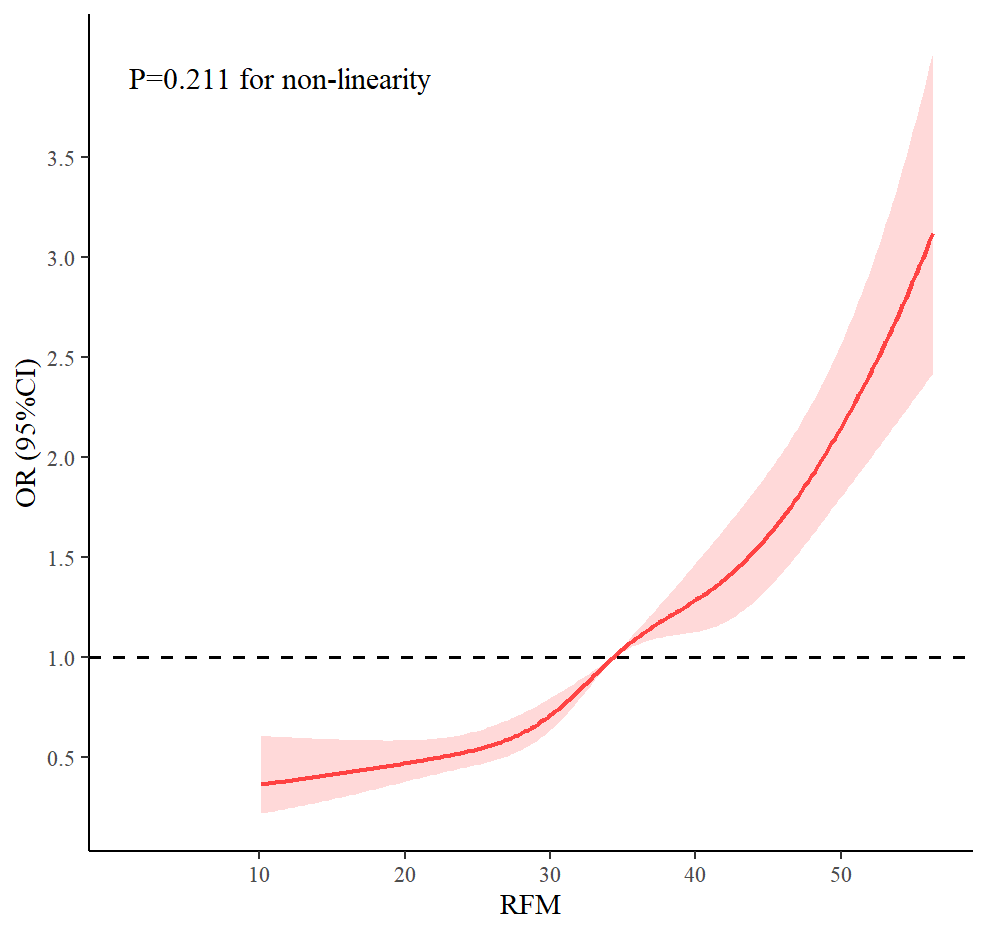


TableS1 Association between RFM and RA(Exclude all missing data)

|  | Model 1 OR (95% CI) P value | Model 2 OR (95% CI) P value | Model 3  OR (95% CI) P value |
| --- | --- | --- | --- |
| RFM | 1.047 (1.040, 1.055) <0.001 | 1.051 (1.038, 1.065) <0.001 | 1.048 (1.035, 1.061) <0.001 |
| Q1(7.756-28.707) | Reference | Reference | Reference |
| Q2(28.707-34.226) | 1.774 (1.428, 2.203) <0.001 | 1.474 (1.180, 1.843) 0.001 | 1.519 (1.211, 1.907) <0.001 |
| Q3(34.226-42.099) | 1.777 (1.436, 2.198) <0.001 | 1.683 (1.289, 2.198) <0.001 | 1.705 (1.302, 2.231) <0.001 |
| Q4(42.099-58.412) | 3.043 (2.487, 3.724) <0.001 | 2.627 (1.939, 3.561) <0.001 | 2.492 (1.839, 3.377) <0.001 |
| P for trend | <0.001 | <0.001 | <0.001 |

RFM=Relative fat mass; RA=Rheumatoid arthritis; OR= Odds ratio; CI=Confidence interval.

Model 1: No covariates were adjusted.

Model 2: Adjusted for age, gender and race.

Model 3: Adjusted for age, gender, race, education level, marital status, Ratio of family income to poverty, smoking status, alcohol consumption.

**Table S2 ROC test results**

| Bootstrap test for comparing correlated ROC curves |
| --- |
| ROC(RFM) vs ROC（BMI） |
| D = 6.3489, boot.n = 2000, boot. stratified = 1, p-value < 2.169e-10 |
